# Evaluation of the α-casein (CSN1S1) locus as a potential target for a site-specific transgene integration

**DOI:** 10.1038/s41598-022-12071-1

**Published:** 2022-05-14

**Authors:** A. V. Smirnov, G. V. Kontsevaya, T. A. Shnaider, A. M. Yunusova, N. A. Feofanova, L. A. Gerlinskaya, I. A. Serova, O. L. Serov, N. R. Battulin

**Affiliations:** 1 Institute of Cytology and Genetics SB RAS, Novosibirsk, Russia 630090; 2 Novosibirsk State University, Novosibirsk, Russia 630090

**Keywords:** Genetic engineering, Gene expression, Transcription, Gene delivery, Gene expression analysis, Genetic engineering, Molecular biology

## Abstract

Transgenic animals are an important tool in biotechnology, including the production of recombinant proteins in the milk. Traditionally, expression constructs are based on hybrid vectors bearing mammary gland specific regulatory elements from the *α-casein* (*Csn1s1*), *β-casein* (*Csn2*), *whey acidic protein* (*WAP*), or *β-lactoglobulin* (*BLG*) genes. Overexpression from the randomly integrated vectors typically provides high levels of expression, but has drawbacks due to unpredictable genome localization. CRISPR-Cas9 targeted transgene integration into the endogenous casein locus could alleviate the need for extensive animal screening to achieve high and reproducible expression levels. We decided to evaluate such a “precise” integration approach, placing the human *granulocyte–macrophage colony-stimulating factor* (*hGMCSF*) gene under control of the mouse endogenous *alpha-S1-casein *(*Csn1s1*) promoter. We designed two types of transgene integrations: a knock-in in the second exon of the *Csn1s1* (INS-GM) and a full-size *Csn1s1* replacement with *hGMCSF* (REP-GM) which was never tested before. The INS-GM approach demonstrated low transgene expression and milk protein levels (0.4% of *Csn2* transcripts; 2–11 µg/ml hGMCSF). This was probably caused by the absence of the 3’-polyadenylation signal in the *hGMCSF* transgene. REP-GM animals displayed high transgene expression, reaching and slightly exceeding the level of the endogenous *Csn1s1* (30–40% of *Csn2* transcripts), but yielded less hGMCSF protein than expected (0.2–0.5 mg/ml vs 25 mg/ml of Csn1s1), indicating that translation of the protein is not optimal. Homozygous inserts leading to the *Csn1s1* knock-out did not have any long standing effects on the animals’ health. Thus, in our experimental design, site-specific transgene integration into the casein locus did not provide any significant advantage over the overexpression approach.

## Introduction

Utilization of the mammary gland specific expression to turn farm animals into bioreactors of valuable proteins has attracted significant attention in the past years^1^. For example, mammary gland directed overexpression of the human recombinant cytokines, such as erythropoietin, granulocyte colony-stimulating factor (hGCSF), granulocyte–macrophage colony-stimulating factor (hGMCSF), or coagulation factors, is highly demanded for pharmacology due to the optimal glycosylation and large protein quantities^2–4^. Most of these approaches has been successfully transferred to farm animals^5–7^.

Clustered regularly interspaced short palindromic repeats (CRISPR)/Cas9-assisted site-specific genome editing is a powerful method for generating transgenic animal lines by pronuclear microinjection^1^. This approach is becoming increasingly popular to create transgenic farm animals with targeted knock-ins in milk modifications^8,9^ (also reviewed in Ref.^10^). However, it is still out-shadowed by the classical random integration approach in milk biotechnology, since the latter shows the highest expression levels (up to g/l) if used with optimal regulatory sequences^11,12^. Unfortunately, random integration of transgenes also carry several inherent flaws, such as potential host genome mutagenesis^13,14^, expression silencing^15,16^ or rearrangements inside the concatenated inserts that frequently contain palindromes^17,18^. These issues could complicate the generation of a predictable and efficient animal producer line. With this in mind, it is important to carefully evaluate the potential of the site-specific single-copy transgene insertions for the future projects.

In theory, the casein locus might be beneficial for endogenous modifications in milk biotechnology as it has complex regulation in lactation^19,20^, that could not be ensured with synthetic constructs of reasonable size. Although some *Csn1s1/Csn2* knock-in experiments were already published^21,22^, the vectors contained supplemental regulatory elements (polyadenylation signals, selection cassettes), which could influence native regulation of the casein locus. The expression from the knock-ins also did not reach endogenous casein levels. We used mouse embryonic stem (ES) cells to create “precise” integrations of the human gene, *hGMCSF*, into the mouse *Csn1s1* open reading frame (ORF) in two variants. The two transgenic models were successfully generated via chimeric mice technology and hGMCSF levels were examined during lactation with enzyme-linked immunosorbent assay (ELISA) and droplet digital PCR. We compared results of our precise integration approach with other published animal models to evaluate its potential.

## Results

### Generation of the transgenic hGMCSF mouse lines

**Figure 1:**
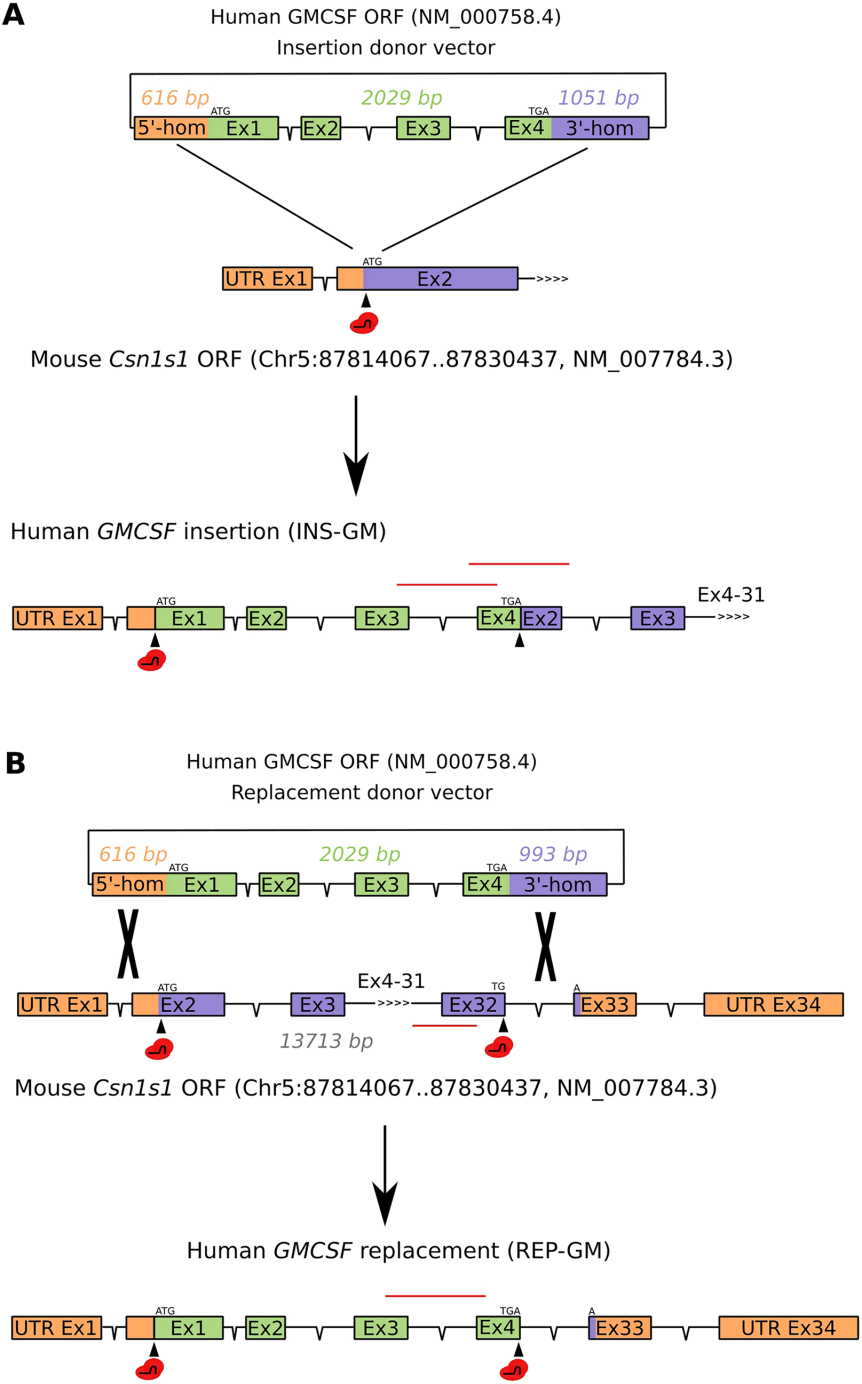
Targeted integration of the *hGMCSF* transgene into the *Csn1s1* locus using CRISPR/Cas9 approach. (**A**) Structure of the *hGMCSF* donor vector and its insertion into the *Csn1s1* Exon 2. (**B**) Replacement of the *Csn1s1* coding sequence with the hGMCSF donor vector. Wild type mouse *Csn1s1* ORF is shown below the vector. Red lines indicate transcript exon-splice sites used for ddPCR expression analysis. Red bubbles symbolize Cas9 cut sites. ATG, TGA—start and stop codons, respectively.

**Figure 2:**
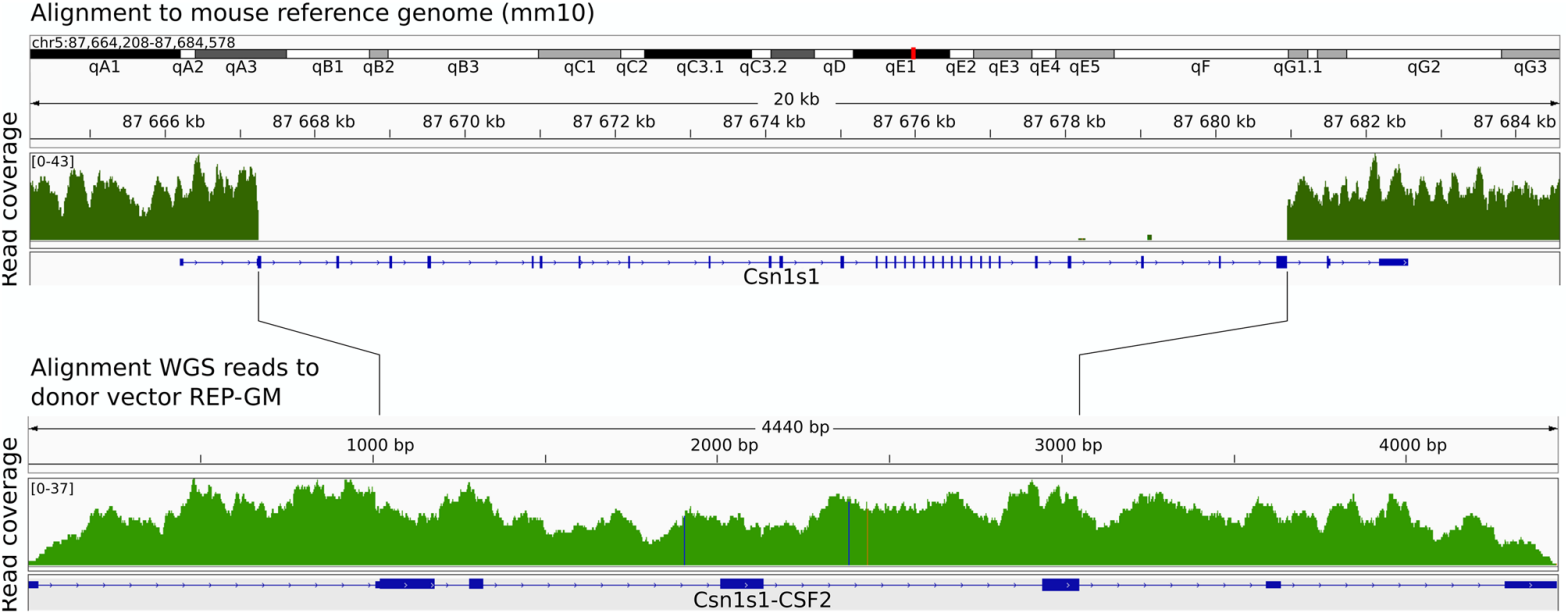
Whole-genome sequencing of the REP-GM homozygous line. Mouse *Csn1s1* locus lost 13.7 Kb of the coding sequence and the *hGMCSF* transgene was inserted instead. Three SNP located at the *hGMCSF* introns were present in the original vector.

We performed targeted integration of the *hGMCSF* open reading frame (ORF) (4 exons, 3 introns, 2029 bp, NM_000758.4) in the casein locus (Fig. 1A). Two types of the *hGMCSF* insertion were tested: (1) a simple knock-in, in which the *hGMCSF* ORF was placed behind the mouse endogenous *Csn1s1* start codon (Fig. 1B, top), while preserving the remaining *Csn1s1* sequence; (2) a complete replacement of the *Csn1s1* ORF (Exons 2–33, 13.7 Kb) with the *hGMCSF* ORF (Fig. 1B, bottom). Genome modifications were carried out in the mouse ES cells with the aid of CRISPR/Cas9 approach. ES cells were transfected with corresponding donor vector (2029 bp *hGMCSF* ORF with 500–1000 bp homology arms), vectors encoding Cas9 protein, guide RNAs (gRNAs) against *Csn1s1*, and a co-selection plasmid (puromycin resistance). Selected ES clones were verified by PCR genotyping (Sup. Figs. 1A, 2A, 7) and Sanger sequencing. In addition, ES clones were characterized by cytogenetic analysis to select clones with > 60% normal 40XY metaphases and less than 10% polyploid metaphases. We have not found any Cas9-associated rearrangements at chromosome 5 Cas9 target sites during routine metaphase examination. ES clones were also screened for off-targets: two off-target sites with the highest scores were analyzed for each guide RNA, and no editing of these sites were observed (Sup. Figs. 5, 6). Of 94 ES clones analyzed, 13 were positive for the correct 5’-border integration event (14%) and 11 of those also showed 3′-border PCR signal, which corresponds to the 85% correct integration (Sup. Fig. 1B, REP-GM). Finally, two transgenic mouse lines (INS-GM, REP-GM) were created by injecting the modified ES clones into recipient blastocysts (Sup. Tables 1, 2). Founders (F0) were crossed with wild-type CD-1 (INS-GM) or C57BL/6J (REP-GM) animals to generate hemizygous F1 animals. Transgene was inherited in Mendelian fashion in both lines (45 and 46% positive pups, respectively). These F1 hemizygous animals were intercrossed to produce homozygous female groups for two transgenic lines. Curiously, there seemed to be some lethality associated with REP-GM modification, because the F2 generation of the F1 × F1 cross had only one homozygous animal (1/37, 3%). Next REP-GM generations showed normal transgene inheritance patterns and were used for lactation experiments. Genome of the homozygous REP-GM line from later generations (F6) was also sequenced with Illumina whole genome sequencing (WGS) to confirm correct transgene integration (Fig. 2) and rule out potential off-target mutations (data not shown) (top ten sites were analyzed for each gRNA in the IGV browser (Sup. Fig. 5, 6)).

### Detection of the human GMCSF in milk of the transgenic mice

We quantitatively measured milk secretion of the hGMCSF using ELISA kit. Milk was collected on day 10 of lactation. Whole milk was diluted with water and assayed on a plate (Sup. Fig. 3) in triplicates. On average, the hGM-CSF milk concentrations constituted 2–11 µg/ml for INS-GM and 161–454 µg/ml for REP-GM (Table 1). In general, REP-GM mice showed much higher expression than INS-GM, and homozygotes had higher levels than hemizygotes (Table 1). Using the same kit, we detected presence of the hGM-CSF in serum of lactating females: the levels were minimal for INS-GM and in a range of 1–3 µg/ml for REL-GM integration.

**Table 1:** ELISA quantification of the hGMCSF in the milk and blood of transgenic female mice.

Transgenic line	Milk hGMCSF levels (µg/ml) ± SD	Blood hGMCSF levels (µg/ml) ± SD
WT 1	0	0
WT 2	0	nd
INS-GM hemi 1	2.4 ± 0.6	nd
INS-GM hemi 2	3.5 ± 0.2	nd
INS-GM homo 1	10.9 ± 1.6	0
INS-GM homo 2	8.5 ± 0.8	nd
REP-GM hemi 1	161 ± 3.0	1.35 ± 0.13
REP-GM hemi 2	212 ± 2.8	nd
REP-GM homo 1	454.8 ± 21.6	2.88 ± 0.08
REP-GM homo 2	298.8 ± 9.7	nd

**Figure 3:**
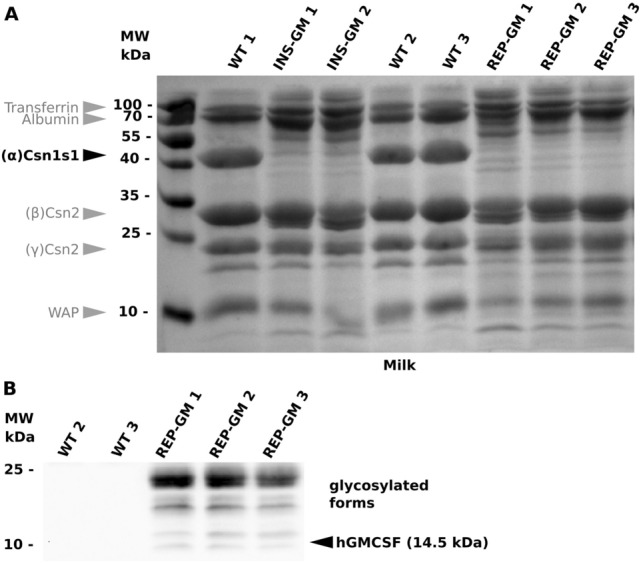
Detection of the hGMCSF in the milk of transgenic mice. (**A**) Total milk protein reversibly stained on a PVDF membrane. WT 1–3—wild-type milk samples; INS-GM 1–2—homozygous INS-GM milk samples; REP-GM 1–3—homozygous REP-GM milk samples. Left lane—protein molecular weight marker (Protein Ladder 10 to 180 kDa). (**B**) Western blot analysis of the REP-GM homozygous milk samples with anti-hGMCSF antibodies.

Diluted milk samples (20 µg of total protein) were separated on SDS-PAGE and transferred to PVDF membrane. The membrane was first stained with a reversible dye to visualize milk proteins (Fig. 3A). As expected, homozygous transgenic lines lacked casein. We also noticed traces of protein degradation, which could be connected to the *Csn1s1* knock-out^23^ (see “Discussion” section). Milk samples were also stained with the anti-hGMCSF antibodies to verify presence of hGMCSF. We were able to detect the band of expected size (14.5 kDa), which had multiple forms due to glycosylation (Fig. 3B, Sup. Figs. 8, 9), similar to previous reports^24^.

**Figure 4:**
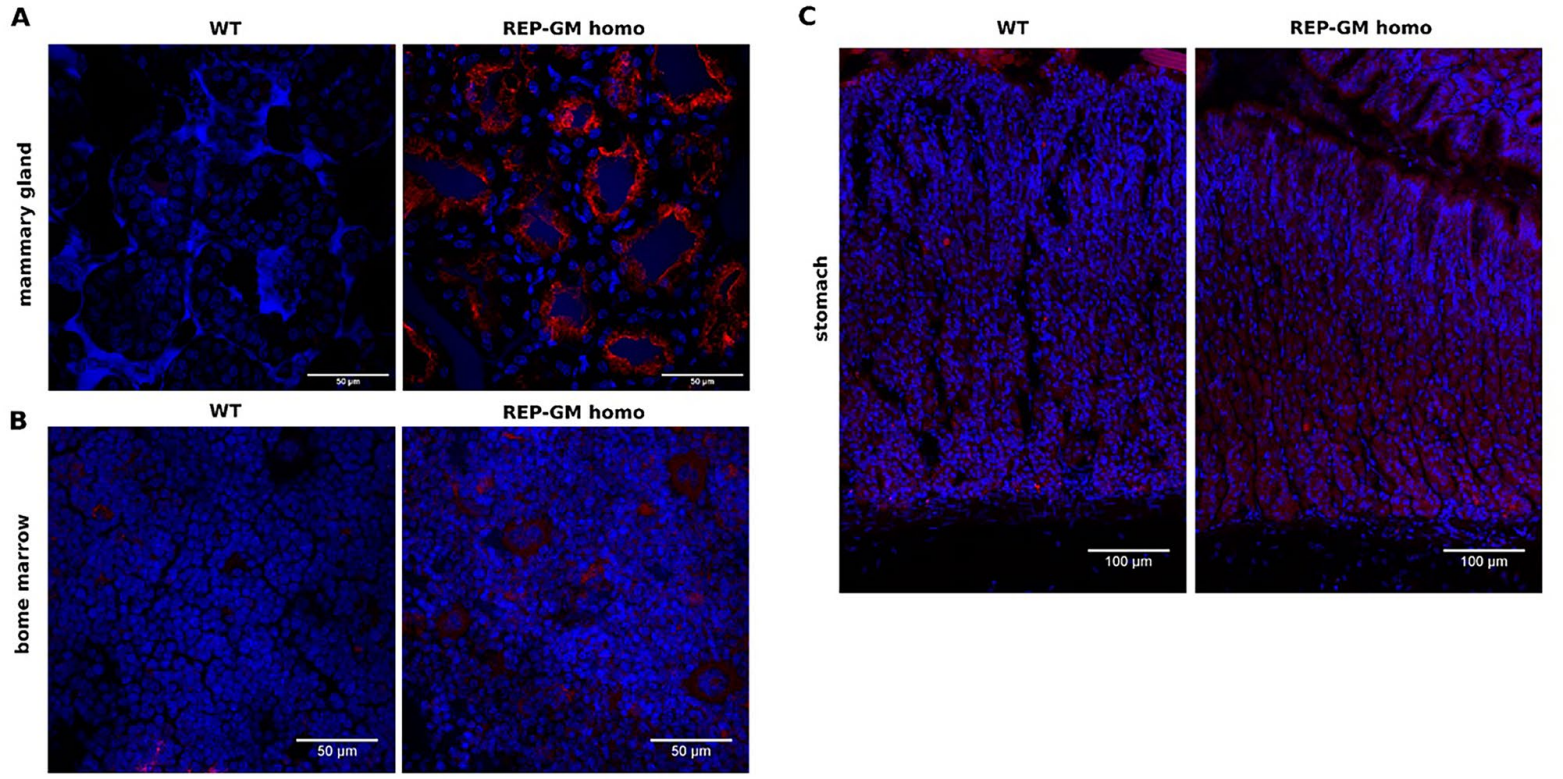
Tissue sections from lactating wild-type and REP-GM homozygous mice stained with anti-GMCSF antibodies (red). Nuclei were stained using DAPI (blue). Scale bars represent 50–100 µm.

Immunohistochemistry staining of mammary gland, bone marrow and stomach cryosections, confirmed the presence of the hGMCSF in the mammary glands of REP-GM mice with no detectable ectopic expression (Fig. 4).

### *Csn1s1* and *hGMCSF* expression analysis by droplet digital PCR

**Figure 5:**
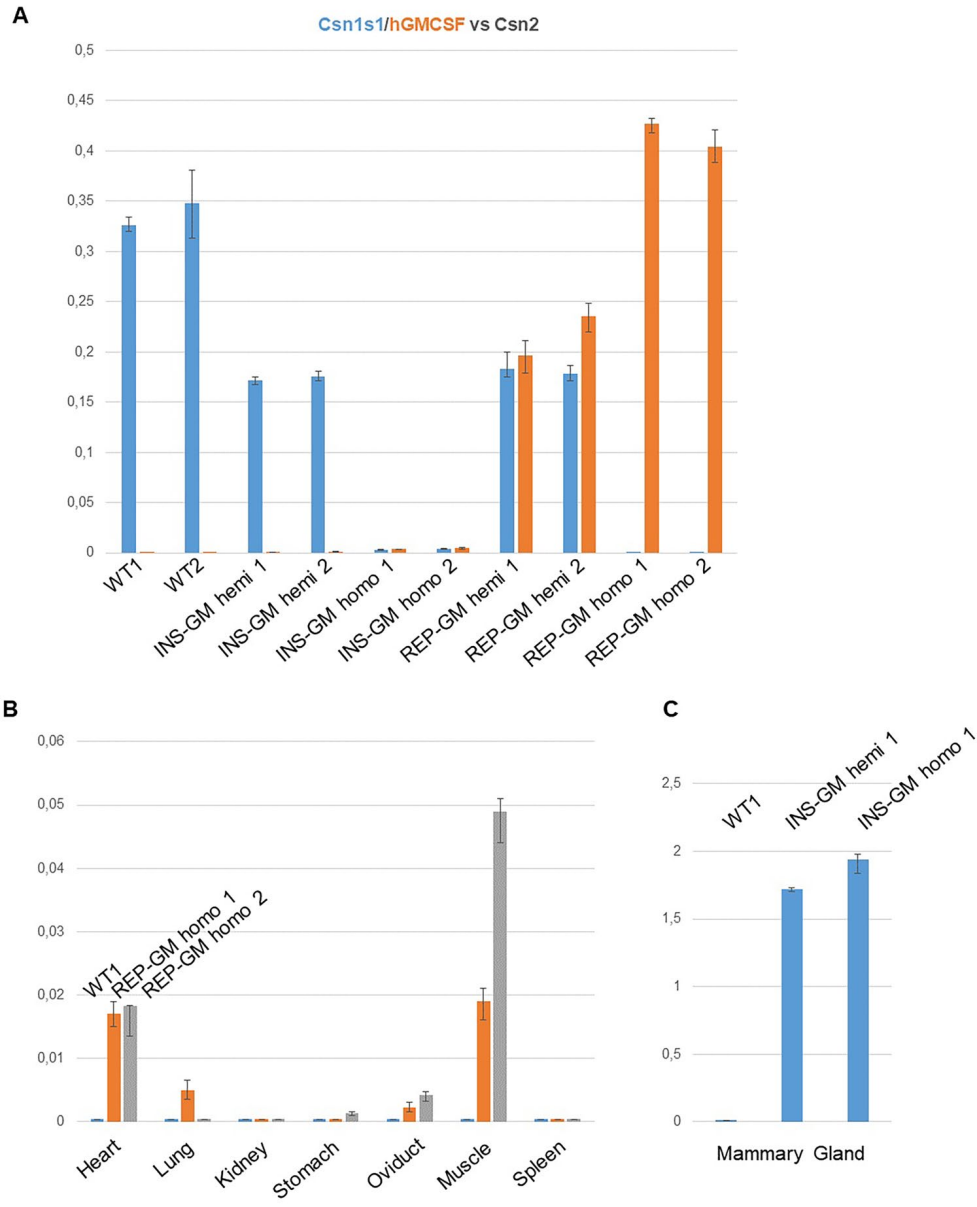
Droplet digital PCR analysis of the *Csn1s1* and *hGMCSF* expression during lactation. Y-axis—relative expression of the target gene vs control gene. (**A**) Ratios of the *Csn1s1* (blue) and *hGMCSF* (orange) transcripts relative to the *Csn2* in mammary glands from various genotypes. (**B**) Evaluation of the *hGMCSF* ectopic expression in various organs, ratios are given relative to the *Rpl4* housekeeping gene. (**C**) Ratios of the *hGMCSF* transcripts relative to *Rpl4* in mammary glands of INS-GM genotypes. Error bars—standard errors.

We anticipated that targeted integration of the transgene would result in a mammary gland restricted expression, similar to the *Csn1s1* gene expression profile. We used droplet digital PCR (ddPCR) to evaluate *hGMCSF* expression across various mouse tissues of lactating females (Fig. 5). Several combinations of probes were tested to analyze *Csn1s1* and *hGMCSF* expression levels. First, we measured *Csn1s1* and *hGMCSF* levels based on the *Csn2* reference (Fig. 5A). To accommodate for the high casein genes expression cDNA was diluted 3000-fold. Interestingly, while expression of the transgene in INS-GM line was very low (~ 0.4% of *Csn2*), REP-GM showed almost identical expression levels of the *Csn1s1* and *hGMCSF* genes in hemizygous mice (Fig. 5A). In homozygous REP-GM animals, levels of the *hGMCSF* were slightly higher than that of the *Csn1s1* in wild-type mice (41% vs 33% of *Csn2*, respectively).

In addition, *hGMCSF* and mouse *Rpl4* gene pair was selected to monitor ectopic expression in various organs (Fig. 5B). Animals from homozygous REP-GM line were chosen for analysis, because they had the highest transgene expression. This time, cDNA was diluted 20-fold. As expected, most of the tissues were completely negative for the *hGMCSF* transcripts, and two samples (heart, muscle) had trace levels of the *hGMCSF* transcripts (Fig. 5B). However, when compared to INS-GM hemizygous and homozygous mice (Fig. 5C), where *hGMCSF:Rpl4* ratio is around 1.5–2, these ectopic expression levels (0.02–0.05) are negligible (REP-GM *hGMSCF:Rpl4* expression difference is too great to compare them directly). Thus, it is safe to assume that the transgene is not expressed outside of the mammary gland in any of the integration variants.

Finally, we studied the *hGMCSF/Csn1s1* chimeric transcript site (exon 4 of *hGMCSF* + exon 2 of *Csn1s1*) (Fig. 1B) in INS-GM. Excision of the *hGMCSF* exons through splicing might have explained impaired expression of the transgene. Levels of the chimeric transcripts were compared to the *Csn2* levels and equaled 0.4–0.6% of *Csn2* transcripts (Sup. Table 3), which was similar to the *hGMCSF* expression based on the internal probe (Fig. 5A, INS-GM homo). Thus, transgene expression was impeded by an alternative molecular mechanism, possibly nonsense-mediated decay.

## Discussion

We have designed two transgene integration variants for the mouse *Csn1s1* locus. Targeted insertion is a simple and promising approach, as such knock-ins could be achieved even in pronuclear microinjection experiments with the aid of CRISPR/Cas9. We anticipated that this integration variant (INS-GM line) would yield high levels of transgene expression, but, conversely, we detected almost no transcription (Fig. 5A). Note that as we tried to preserve native regulatory landscape, we did not include the polyadenylation signal into the transgene’s 3′-region. This might have caused nonsense mediated decay (NMD), which facilitated degradation of the transcripts due to premature stop codon^25,26^. Other similar modifications of the casein or another milk genes using polyadenylation signals reported various levels of expression. For instance, integration of the porcine *lactoferrin* into exon 17 of porcine *Csn1s1* in pigs showed high levels of expression^21^. Knock-ins of the human *lactoferrin* and *α-lactalbumin* into the goat β-*lactoglobulin* (*BLG*) gene resulted in high expression in range of ~ 1–3 mg/ml^27,28^; while, cows with the human lysostaphin knock-in to endogenous *Csn2* exon 2 had only moderate protein levels (less than 10 µg/ml)^22^. Our own replacement variant (REP-GM) which uses the same *Csn1s1* promoter and transgene ORF, but relies on the *Csn1s1* 3′-UTR (Fig. 5A) demonstrated much higher expression than the INS-GM. Moral of the story: always use polyadenylation signals in knock-ins.

Transgene replacement approach (REP-GM) provided high *hGMCSF* expression in homozygous animals (Table 1). Importantly, *hGMCSF* gene expression was equal to the endogenous *Csn1s1* allele in hemizygous mice as were discovered by a sensitive ddPCR assay (Fig. 5A). This means that in contrast to the INS-GM line, transcriptional regulation of the locus was preserved in the native state. Yet, the protein concentration (0.5 mg/ml at max) in milk was much lower than could be expected extrapolating from the endogenous Csn1s1 levels (ca. 25 mg/ml in mice)^23,29^. We cannot explain the discrepancy between *hGMCSF* mRNA and protein levels, but it is apparently caused either by hGMCSF leakage through secretion pathways or inefficient translation. One of the most plausible reasons is an improper signal peptide. We made a full size replacement of the *Csn1s1* ORF from start to stop codon and, as a consequence, the N-terminal Csn1s1 signal peptide (1–15 aa) was replaced with the N-terminal hGMCSF secretion signal (1–17 aa). It is known that native signal peptide could not compete in efficiency with the milk-specific secretion signals, such as *BLG* N-terminal fragment (1–18 aa) which could improve recombinant protein production by several orders^30^. We have tried to generate *hGMCSF* transgenic mouse line with preserved Csn1s1 signal peptide through pronuclear microinjection, using the same CRIPSR/Cas9 approach (donor vector + gRNA + Cas9) we conducted in ES cells (Fig. 1). Unfortunately, we did not detect successful integration events, although 90% of the pups had mutations at the *Csn1s1* site^31^. Thus, additional studies are welcome to understand if preservation of the Csn1s1 signal peptide may be a key to sustain the highest recombinant protein levels during gene targeting.

We compared the observed hGMCSF expression levels with other mammary gland specific overexpression experiments in mice and farm animals (exact numbers and transgene descriptions could be found in thematic reviews^10,32,33^). Numerous studies reported very high recombinant protein levels (up to 1–30 mg/ml) in milk, which seems to be a maximum limit of secretion^11,12,34,35^. At the same time, a comparable number of reports showed modest levels of recombinant proteins within µg/ml^24,36^ or even ng/ml ranges^8,37^. Clearly, the expression efficiency is strongly dependent on the vector structure, and pBC1 commercial vector or other hybrid promoter/enhancer vectors show the best results^12,38^. In our experimental approach with no added regulatory sequences and native secretion signal, the *Csn1s1* replacement strategy demonstrated rather “satisfactory” hGMCSF levels (0.2–0.5 mg/ml).

It is important to consider potential effects of gene deficiency when producing homozygous animals with site-specific knock-ins. In our case, both lines had functionally inactive *Csn1s1* gene (Figs. 3A, 5A). In contrast to the non-essential *Csn2* gene^39^, the *Csn1s1* knockout has some effects on milk composition as studied in detail by Ref.^23^.

First, the *Csn1s1* knock-out might result in the *Csn2* expression downregulation^23^. Authors claim that in their gene-trap model, where a cassette integrated into the second exon of the *Csn1s1*, expression of the other casein genes (*Csn2*, *Csn3*) dropped below 20%. Although the casein locus expression regulation is studied in detail and potential enhancer interactions are mapped^20^, no molecular mechanisms have been suggested to explain this observation. Original authors suggest that these might be caused by endoplasmic reticulum stress^23^. Either way, independent confirmation and understanding of this phenomenon would be important for future projects with the casein-targeting knock-ins. It seems that additional stage-specific RNAseq experiments are required to investigate how knock-outs of the casein genes affect the locus expression during lactation. Although our transgenic lines could potentially serve for this purpose, it is desirable to create CRISPR/Cas-directed precise casein modifications, such as frame-shift mutations, ORF deletions, or large deletions in the casein locus.

Same report also stated that the Csn1s1 deficiency leads to degradation of milk protein components^23^. This effect is probably caused by milk micelle destabilization, because Csn1s1 plays a crucial role in its membrane. We have noticed some milk protein fragmentation patterns on our gels in homozygous animals (Fig. 3A), but additional confirmation is required to validate it. It is well established that overexpression of the heterologous proteins induces general endoplasmic reticulum (ER) stress, including impairments of protein folding homeostasis and protein degradation machinery, which in turn causes protein stability issues. It would be extremely interesting to study how these two combined factors (ER stress + Csn1s1 KO) affect recombinant protein stability. If this is the case, it would be required to perform double protein purification using N- and C-terminal tags or avoid breeding homozygous animals for protein production.

Finally, pups nurtured by the Csn1s1-deficient mothers showed slower growth during the first month of life, due to deficient nutrition (both INS-GM and REP-GM lines). This growth lag was only temporary and did not affect general development and fertility, as was described before^23^.

In conclusion, precise site-specific integration of transgenes into the Csn1s1 does not seem especially beneficial. On the one hand, it may be useful to generate animal bioreactors with predictable and stable tissue-specific expression. However, transgenic animals with overexpression from the randomly integrated hybrid vectors yield higher protein levels while being much simpler in the making. Applications of the CRISPR technologies to the generation of multiple knock-in animals will be necessary to systematically assess potential of milk loci targeting for biotechnology, including alterations in the transgenic ORF design, such as heterologous signal peptides and synthetic polyadenylation signals.

## Methods

### Construction of the donor vectors and modifications of mESCs

Donor vectors for insertion/replacement modifications were based on the OCT4-eGFP-PGK-Puro vector (Addgene #31937). PGK-Puro cassette was removed to exclude undesired effects on the native regulation of the Csn1s1 gene after integration. Homology arms were inserted consequently with Gibson cloning. Csn1s1 homology regions for insertion vector were: Chr5: 87,814,490–87,815,105 (616 bp) (5′-arm) and Chr5: 87,815,135–87,816,185 (1051 bp) (3′-arm); for replacement: Chr5: 87,814,490–87,815,105 (616 bp) (5′-arm) and Chr5: 87,828,822–87,829,814 (993 bp) (3′-arm).

Mouse embryonic stem cells (mESCs, DGES1 cell line, 129S2/SvPasCrl) were transfected with corresponding donor vectors (insertion/replacement), Puro^R^ co-selection plasmid, Cas9 (Addgene #41815) and gRNA-expressing plasmids (Addgene #41824, backbone) using Lipofectamine 2000. Sequences of gRNAs are provided in Sup. Figs. 5 and 6. Selection of the positive clones was performed using 2 μg/ml puromycin for 2 days. All cell lines were grown at 37 °C with 5% CO_2_ and passaged every 2–3 days. Surviving ES colonies were subcloned and PCR genotyped using primers from Sup. Table 4. PCR-positive clones were examined by cytogenetic screening to select those with normal karyotypes > 60% (10–20 metaphases per ES clone). Two selected clones with hGMCSF integration (INS-GM and REP-GM) were additionally analyzed with Sanger sequencing of homology arms, hGMCSF ORF and top off-target sites before the generation of chimeric animals.

The embryonic stem cell lines used in this work are available at a Collective Center of ICG SB RAS Collection of Pluripotent Human and Mammalian Cell Cultures for Biological and Biomedical Research (https://ckp.icgen.ru/cells/; http://www.biores.cytogen.ru/brc_cells/collections/ICG_SB_RAS_CELL).

### Producing transgenic mouse lines via chimeric animals

Selected ES clones were plated on gelatin and cultured for 3 passages after thawing in standard ES cell 2i medium (1 μM PD, 3 μM CHIR, DMEM (ThermoFisher), 7.5% ES FBS (Gibco), 7.5% KSR (Gibco), 1 mM l-glutamine (Sigma), NEAA (Gibco), 0.1 mM β‐mercaptoethanol, LIF (1000 U/ml, Polygen), penicillin/streptomycin (100 U ml—1 each)).

CD-1 or B6D2F1 blastocysts from the superovulated females were collected at day 3.5 post coitus, washed out with M2 medium (Sigma-Aldrich) and placed in KSOR medium (CosmoBio) covered with mineral oil at 37 °C, and injected with 10–15 cells under a microscope (TransferTip, Eppendorf). Blastocysts were monitored in KSOM medium for 2 h at 37 °C, 5% CO2. Surviving blastocysts were transplanted to pseudopregnant CD-1 females (12–13 per female). F0 males with a high percentage of chimerism were crossed with CD1 or C57BL/6J females (Sup. Table 3, 4). Agouti-colored F1 pups were genotyped with primers for the transgene’s 5′-homology arm (Sup. Fig. 1). F1 animals were crossed to produce F2 homozygous females for milk analysis. To discriminate between hemizygous and homozygous hGMCSF inserts, primers for transgene’s 5′-homology arm and for mouse Csn1s1 were used (Sup. Fig. 1, Sup. Table 4).

Animals were kept in a standard environment at 24 °C temperature, 40–50% relative air humidity and 14 h light/10 h dark–light-cycle. Food and water were available ad libitum. Lactating mice were euthanized by isoflurane inhalation immediately after milk collection. At the end of experiments, remaining animals were euthanized by CO2. All experiments were conducted at the Centre for Genetic Resources of Laboratory Animals at the Institute of Cytology and Genetics, SB RAS (RFMEFI61914X0005 and RFMEFI61914X0010). All experiments were performed in accordance with protocols and guidelines approved by the Animal Care and Use Committee Federal Research Centre of the Institute of Cytology and Genetics, SB RAS operating under standards set by regulations documents Federal Health Ministry (2010/708n/RF), NRC and FELASA recommendations. Experimental protocols and euthanasia procedures were approved by the Bioethics Review Committee of the Institute of Cytology and Genetics. The manuscript followed the recommendations in the ARRIVE guidelines.

### Milk collection and analysis

Milk was collected with a pipette from narcotized female mice at day 10 of lactation after oxytocin administration and stored at − 80 °C. For ELISA analysis, milk was diluted 1–6 million times with water and assayed according to the manufacturer’s recommendation (Human GM-CSF Quantikine ELISA Kit, DGM00, R&D Systems). Blood was collected from the euthanized mice and the serum was diluted × 1000 times for the assay. Measurements were taken at 490 nm with BioTek Epoch Spectrophotometer.

The protein concentrations in milk were quantified using Pierce BCA Protein Assay Kit (ThermoFisher). For Western blot, equal amounts (20 μg) of milk samples were separated on 15% SDS-PAGE, and then transferred onto Immun-Blot PVDF membrane (Bio-Rad). Membrane was blocked with 5% milk/TBST(20 mM Tris pH 7.5, 150 mM NaCl, 0.1% Tween 20) for 2 h and incubated with primary antibodies against hGMCSF 1:500 at 4 °C overnight (R&D, catalogue # AF-215-NA). Next day, membranes were incubated with horseradish peroxidase-conjugated secondary antibodies 1:1000 (#93702, Cell Signaling) for 2 h at 25 °C. Detection was performed with SuperSignal West Pico PLUS Chemiluminescent Substrate (#34580, ThermoFisher) and Chemidoc XRS Imaging system (Bio-Rad). Reversible staining of the PVDF membrane (Fig. 3A) was performed with Pierce Reversible Protein Stain Kit (#24585, ThermoFisher).

### RNA extraction and ddPCR analysis of expression

Total cellular RNA was extracted from mouse mammary glands and other organs in glass homogenizers using TRI Reagent (Sigma-Aldrich). 1 μg of total RNA was used to generate cDNA in a 20 μl reaction using RevertAid RT Kit (Thermo Fisher Scientific) with random hexamer primers according to the manufacturer’s instructions. Droplet digital PCR (ddPCR) was performed using a QX100 system (Bio-Rad) with primers and probes specific for the *Csn1s1, Csn2, Rpl4, hGMCSF,* and *hGMCSF/Csn1s1* chimeric transcripts. The primers and probes sequences are presented in Sup. Table 4. ddPCR reactions were set in 20 μl volumes containing 1× ddPCR Supermix for Probes (no dUTP), 900 nM primers and 250 nM probes, and 1 μl of 20- or 3000-fold diluted cDNA or 20 ng DNA. ddPCR reactions for each sample were performed in duplicates. PCR was conducted according to the following program: 95 °C for 10 min, then 41 cycles of 95 °C for 30 s and 61 °C for 1 min, with a ramp rate of 2 °C per second, and a final step at 98 °C for 5 min. The results were analyzed using QuantaSoft software (Bio-Rad). Thresholds were set to 6000 for the *Csn1s1, Csn2, Rpl4, hGMCSFxCsn1s1* genes (Sup. Fig. 4); 4500 for the *hGMCSF* gene (Sup. Fig. 4B); 5000 for the *Emid1* and *hGMCSF* DNA regions (Sup. Fig. 2B).

### Immunohistochemistry

Intracardiac perfusion of euthanized mice was performed manually with 4% PFA solution in 1× PBS. Organs were collected in 1× PBS and fixed in 4% PFA solution overnight on a roller shaker at 4 °C. Next day samples were washed in 1× PBS solution three times for 30 min. For tissue dehydration organs were sequentially incubated for at least 24 h with a 15% and 30% sucrose solution in 1× PBS at 4 °C. Next, organs were embedded in Tissue-Tek O.C.T. Compound (Sakura Finetek) and frozen. Organ sections of 50 um thickness were prepared on MICROM HM 505N cryostat (Microm) and immediately collected on SuperFrost™ slides (Thermo Scientific). Sections were washed with 1× PBS and incubated in blocking solution: 2% BSA (Sigma Aldrich), 0.2% Triton X-100 (Amresco), 5% FBS (Capricorn Scientific). Primary antibodies against human GMCSF (R&D, catalogue # AF-215-NA) were diluted in blocking solution and incubated with sections overnight at slow speed on an orbital shaker at room temperature. Next, slices were washed with 1× PBS three times for 20 min and stained with secondary antibodies (Jackson Immuno Research, catalogue # 705-165-147) and DAPI diluted in 1× PBS for 2 h at room temperature. Slices were washed with 1× PBS three times for 20 min and completely dried out and finally were mounted with ProLong™ Diamond Antifade Mountant (Thermo Fisher Scientific). Immunofluorescence was visualized under confocal fluorescence microscope LSM 780 NLO (Zeiss) with ZEN software (Zeiss).

### Whole genome sequencing

Genomic DNA was extracted from the tail tip of a homozygous REP-GM animal and subjected to paired-end 2 × 150 bp Illumina sequencing, generating approximately 317 million reads. Raw reads were analyzed using FastQC software to ensure high data quality and mapped to the mouse genome (mm10) with Bowtie 2 with default parameters. To perform data visualization, alignments were converted to BAM format, sorted and indexed using SAMtools, and loaded as the IGV browser track. For off-target mutations analysis, selected off-target sites (Sup. Fig. 5, 6) were manually inspected in the IGV browser.

## Supplementary Information

Supplementary Information.

## Data Availability

WGS sequencing data can be accessed online at Sequence Read Archive under the BioProject accession code PRJNA772364.
