# Supplementary material for: Evaluation of the α-casein (CSN1S1) locus as a potential target for a site-specific transgene integration
Source: Sci Rep. 2022 May 14;12:7983. doi: 10.1038/s41598-022-12071-1 (PMC9107462; doi:10.1038/s41598-022-12071-1)
Supplement: Supplementary file 1 — Supplementary Information. [file 41598_2022_12071_MOESM1_ESM.pdf]

SUPPLEMENTARY FIGURES

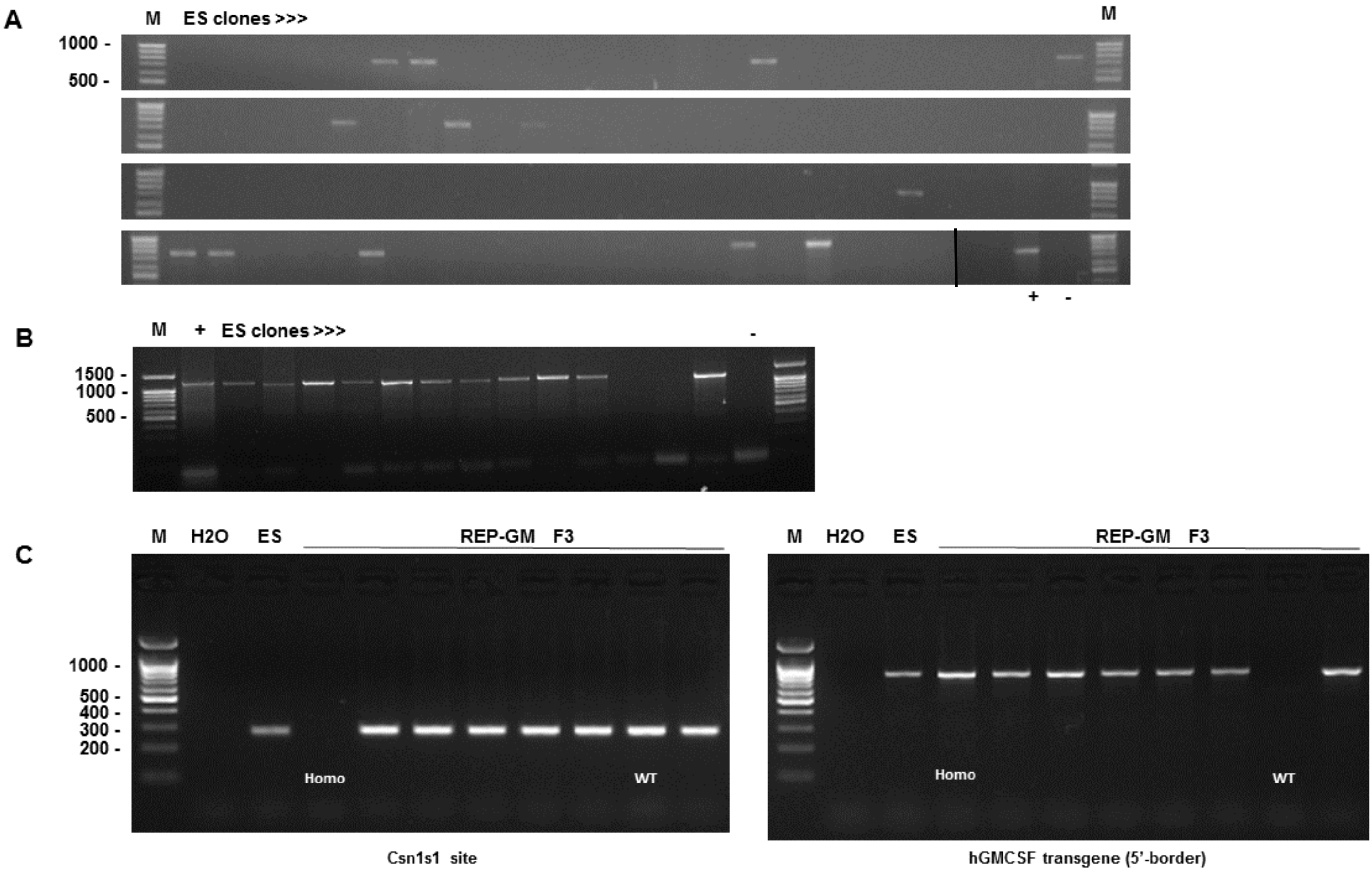

Supplementary Figure 1. PCR genotyping of the ES clones and transgenic mice with REP-GM integration variant. (A) Initial genotyping of the ES clones that survived after puromycin selection. Primers at the Csn1s1-hGMCSF 5'-border were used to detect positive clones (781 bp). (B) Additional examination of the 3'-border to verify correct integration of the construct (only in the initially positive clones). Primers at the Csn1s1-hGMCSF 3'-border were used to detect correct ES clones (1206 bp). One of the clones was selected for generation of the transgenic animals. (C) Genotyping of the transgenic offspring from the REP-GM line with primers for Csn1s1 (283 bp) and hGMCSF (781 bp). Legend: M - 100 bp ladder; "+" - positive control; "-" or H2O - negative control; ES - original ES clone; REP-GM F3 - animals for genotyping.

A

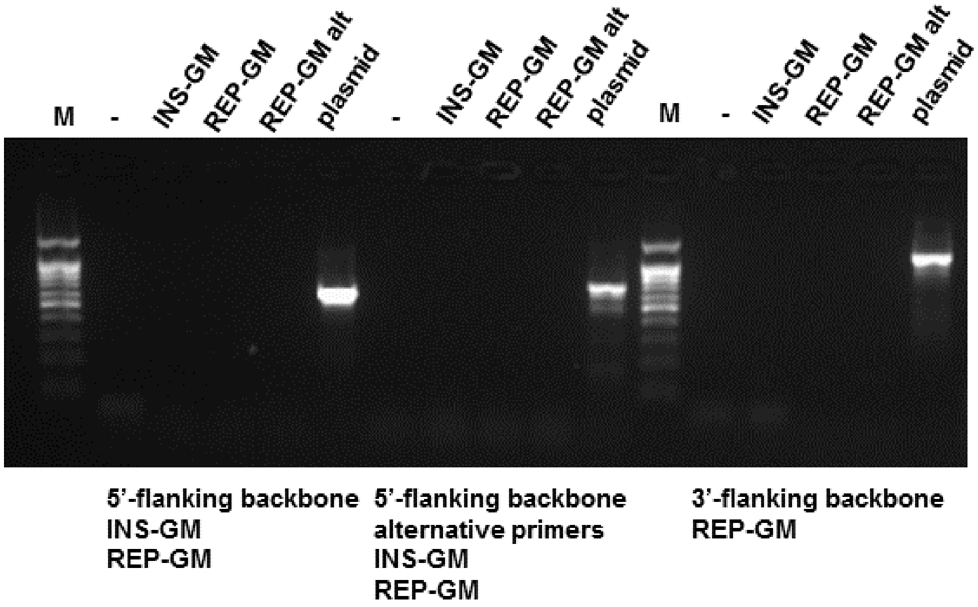

B

Genotype	Copy number hGMCSF vs Emid1	Standard error
WT	0	0
ES INS-GM	0,851	0,718-0,985
ES REP-GM	0,899	0,859-0,938
INS-GM hemi 1	0,887	0,846-0,929
INS-GM hemi 2	0,954	0,921-0,987
REP-GM hemi 1	0,815	0,752-0,885
REP-GM hemi 2	0,939	0,906-0,972
INS-GM homo 1	1,85	1,8-1,9
INS-GM homo 2	1,79	1,74-1,84
REP-GM homo 1	1,83	1,78-1,89

Supplementary Figure 2. Confirming an absence of the randomly integrated transgenes. (A) PCR for the backbone regions of the vectors in selected ES clones. M – marker 100 bp; INS-GM and REP-GM – ES cells used for generation of transgenic animals; REP-GM alt – additional ES clone; plasmid – a mix of two donor vectors for both insertion variants; (-) – negative control. (B) Quantification of the transgene copy number (hGMCSF) in ES clones and F2 animals with ddPCR. Emid1 was used as a reference gene (CNV2).

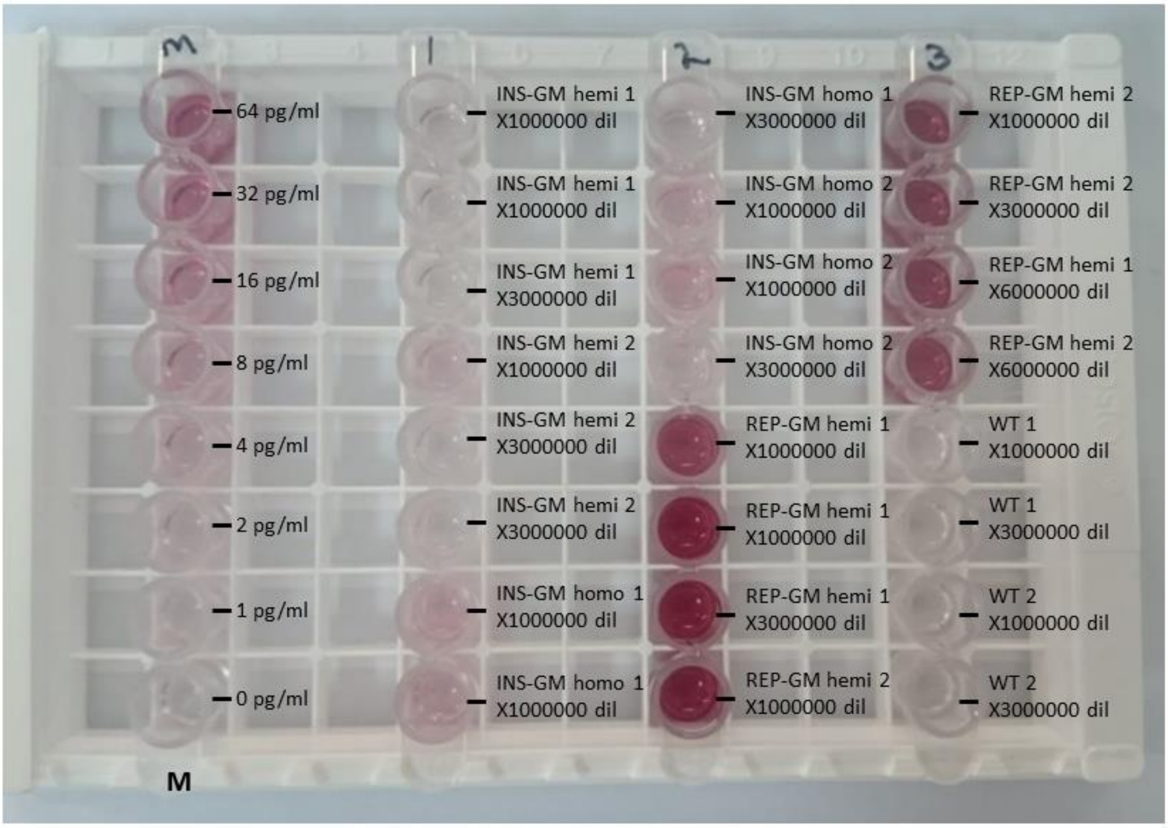

Supplementary Figure 3. Photograph of a representative ELISA assay for diluted milk samples Human GM-CSF Quantikine ELISA Kit DGM00, R&D Systems). M – protein standards of the hGMCSF. The absorbance was determined in a spectrophotometer at 490 nm.

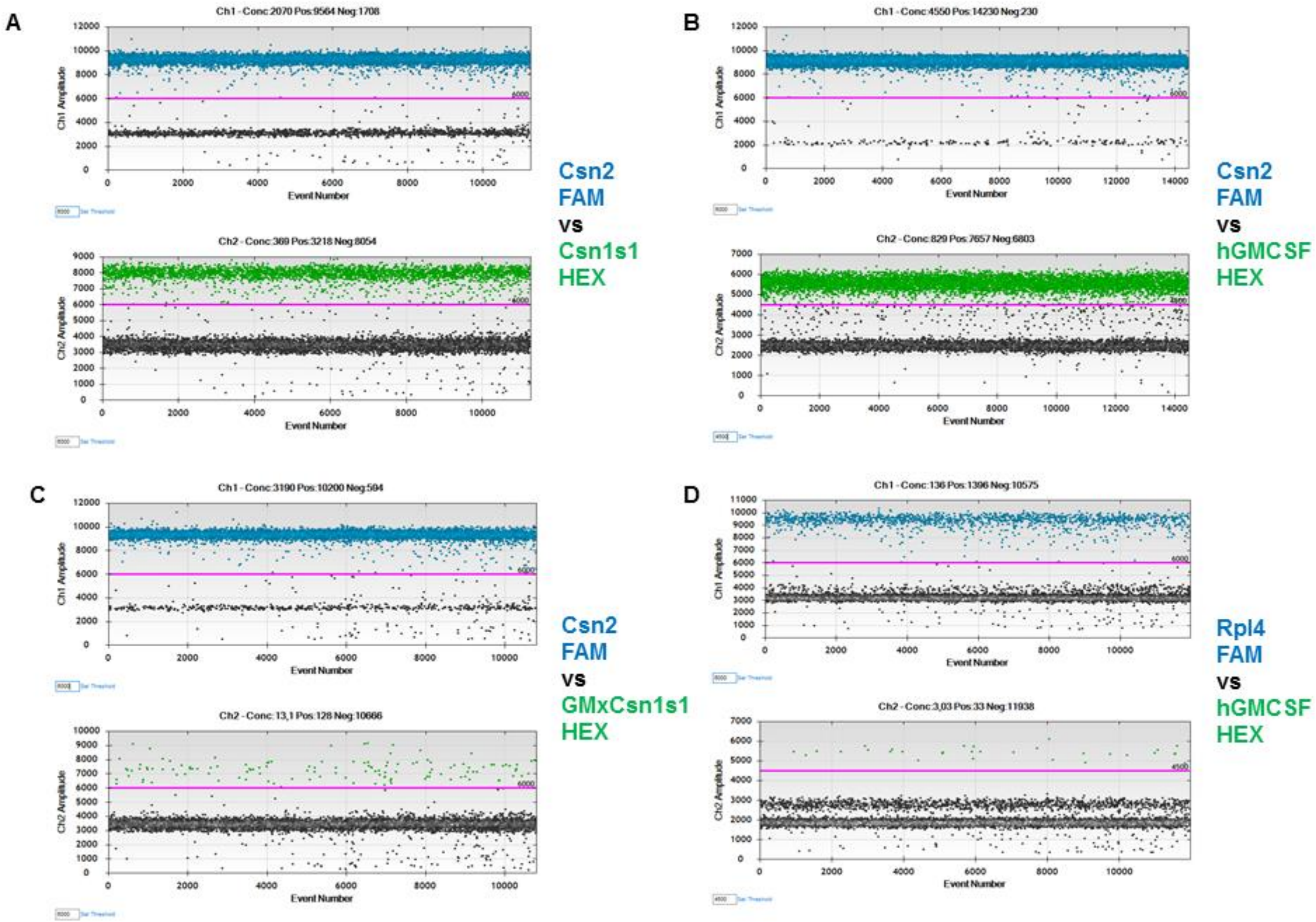

Supplementary Figure 4. Representative ddPCR plots for the assays used in the project. (A-D) Pairs of the analyzed genes (blue/green).

gRNA-1 (*Csn1s1* ATG) off-targets

Off-target Site with PAM	Score	Location	Strand
GGTGAGGATGAGGAGTTTCA-TGG	100	Chr5:87667251 (*Csn1s1*)	(-)
GGTGAGGATGGGGAGTTTCA-GAG	55.5	Chr1:24388301	(+)
GGTGGGGATGGGGAGTTTCA-GAG	3.7	Chr4:140044179	(-)
CTTGAGGATGGGGAGTTTCA-CAG	1.7	Chr10:115257073	(-)
AATTAGGATTAGGAGTTTCA-GAG	1.4	Chr19:7026799	(+)
GCTTTGGATCAGGAGTTTCA-TAG	1.4	Chr4:93995499	(-)
CCAGTGGATGAGGAGTTTCA-AAG	1.4	Chr8:68556410	(+)
CTTGAGGATGAGGAATTTCA-TGG	1.0	Chr3:8252750	(+)
TGTCAGGTAGAGGAGTTTCA-TGG	1.0	Chr15:72538325	(+)
ATCGAGGAAGAGGAGTTTCA-CAG	0.9	ChrX:70147892	(+)
AGTGGCGGTGAGGAGTTTCA-TGG	0.9	Chr1:82926315	(-)

gRNA-1 (*Csn1s1* ATG) – off-target 1 (Chr1:24388301)

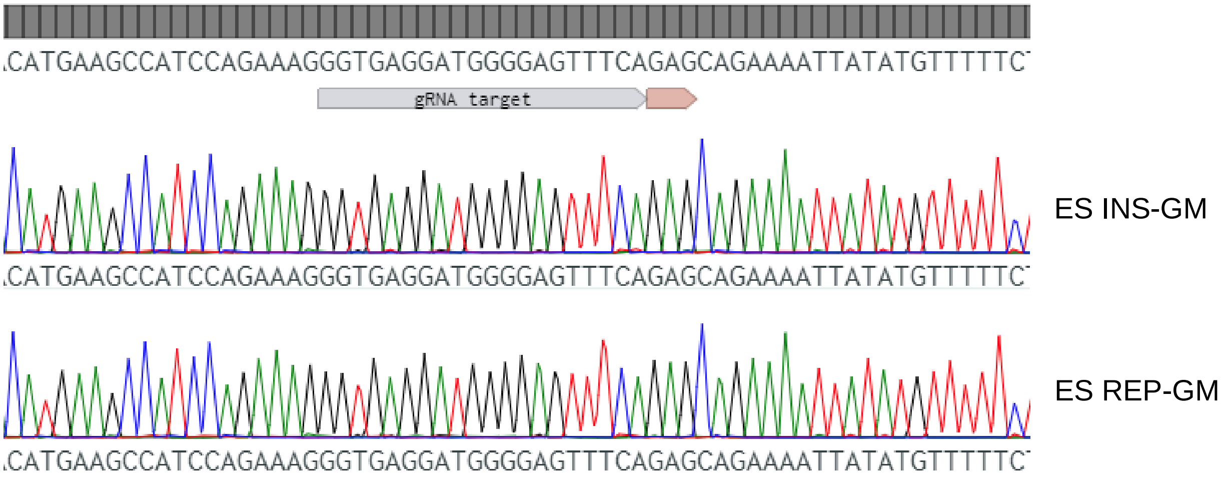

gRNA-1 (*Csn1s1* ATG) – off-target 2 (Chr4:140044179)

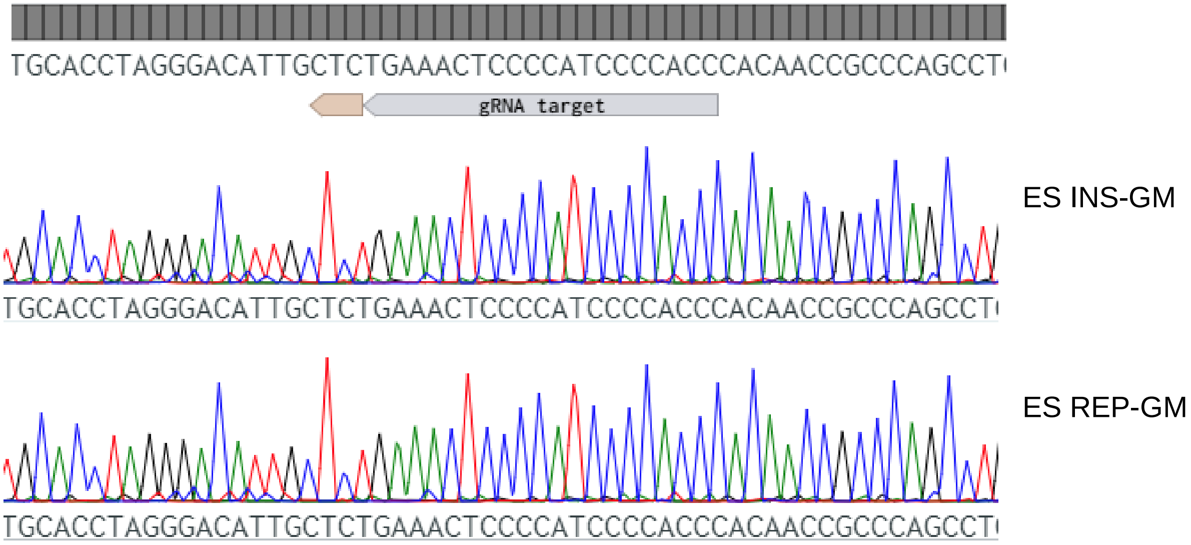

Supplementary Figure 5. List of top 10 off-target sites for gRNA-1, scoring based on (Hsu et al., 2013), benchling.com. Higher score reflects increased chance of Cas9 cleavage. Two off-target

sites with the highest score were Sanger sequenced to confirm absence of mutations in the donor ES cells (INS-GM, REP-GM).

gRNA-2 (*Csn1s1* 3'-UTR) off-targets

Off-target Site with PAM	Score	Location	Strand
CTGAAAAAACCAATGTGTGG-TGG	100	Chr5:87680958 (*Csn1s1*)	(+)
CTGACCAAACCAATGTGTGG-TGG	3.2	ChrX:152145355	(-)
TTGTTAAAACCAATGTGTGG-GAG	2.5	Chr12:106326578	(-)
CTGACAACAGCAATGTGTGG-AAG	2.4	Chr9:50332929	(-)
ATGTAAAAACCTATGTGTGG-TAG	1.6	Chr14:36457557	(-)
GTGATAACAGCAATGTGTGG-CAG	1.5	Chr9:115518077	(+)
AAGCAAAAAGCAATGTGTGG-GAG	1.5	Chr12:20887740	(+)
CTGAGGAAATCAATGTGTGG-AGG	1.4	Chr4:151450166	(+)
GTTTAAAAAGCAATGTGTGG-AGG	1.4	Chr2:60762761	(+)
ATAGCAAAACCAATGTGTGG-GGG	1.4	Chr2:158167975	(-)
TTGAAAATACCACTGTGTGG-GAG	1.3	Chr16:93893982 (*Chaf1b*)	(+)

gRNA-2 (*Csn1s1* 3'-UTR) – off-target 1 (ChrX:152145355)

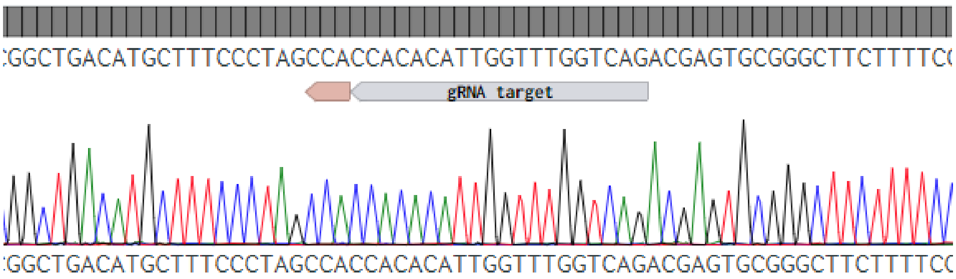

ES REP-GM

gRNA-2 (*Csn1s1* 3'-UTR) – off-target 2 (Chr12:106326578)

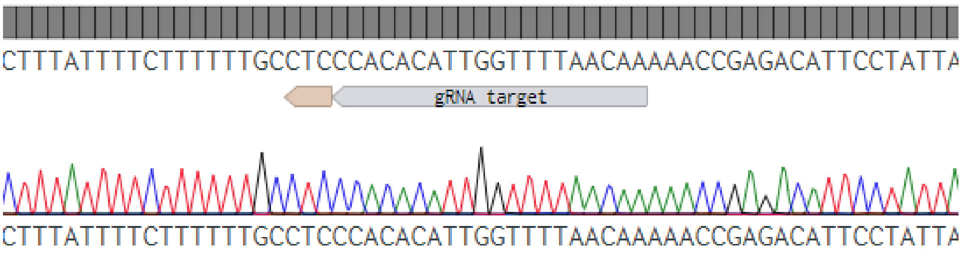

ES REP-GM

Supplementary Figure 6. List of top 10 off-target sites for gRNA-2, scoring based on (Hsu et al., 2013), benchling.com. Higher score reflects increased chance of Cas9 cleavage. Two off-target sites with the highest score were Sanger sequenced to confirm absence of mutations in the donor ES cells (REP-GM).

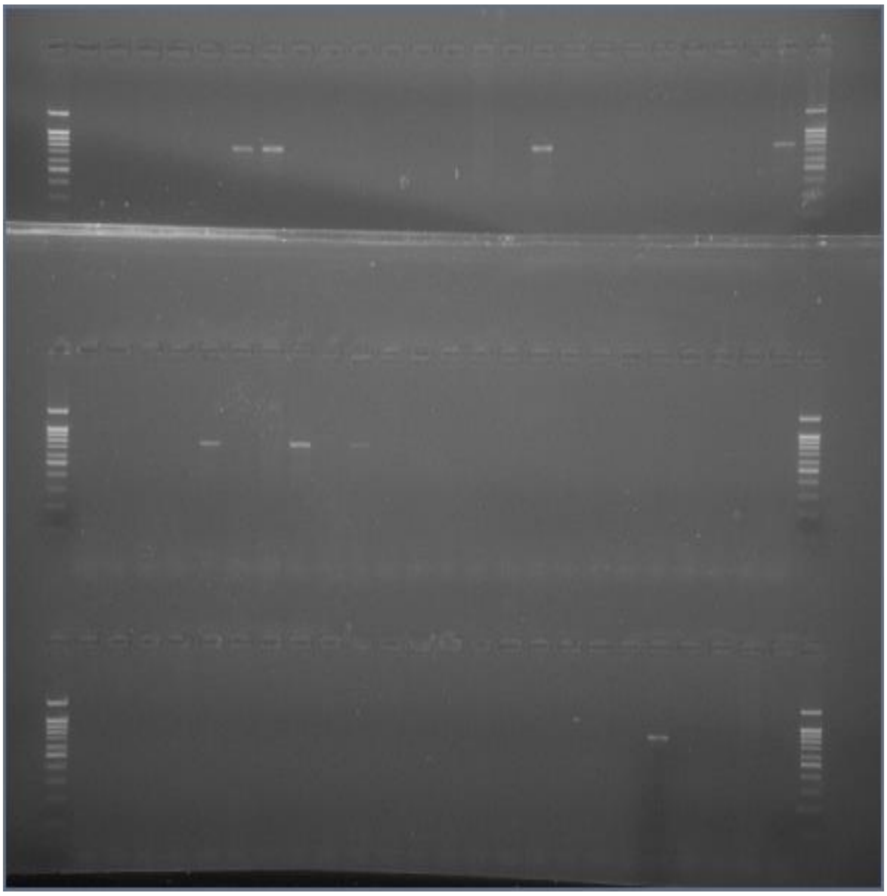

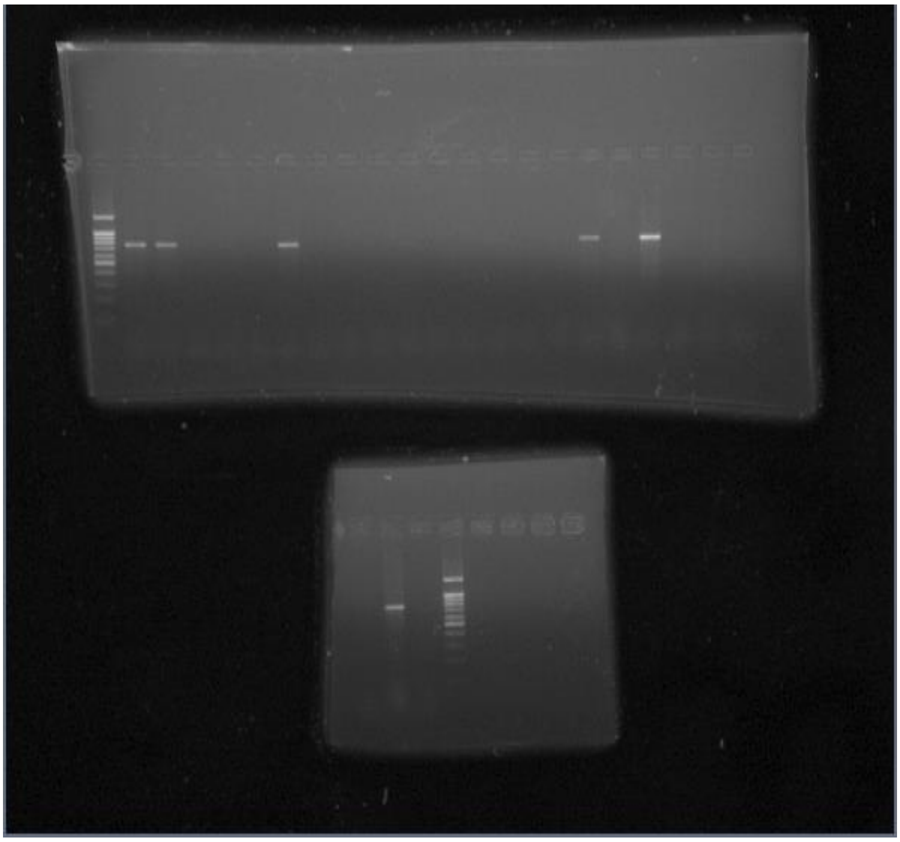

Supplementary Figure 7. Unedited agarose gels for PCR genotyping (Sup. Fig. 1)

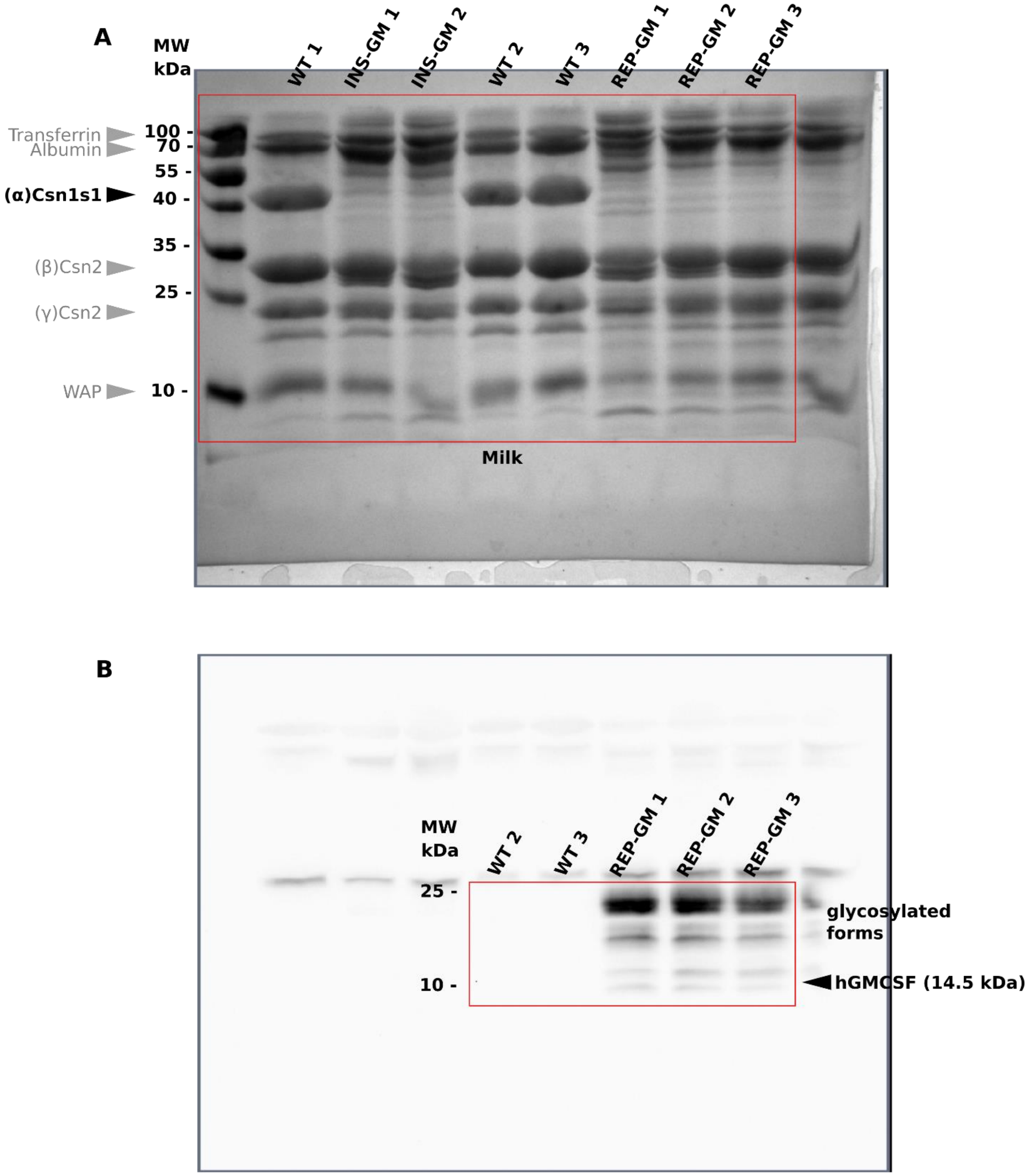

Supplementary Figure 8. Unedited SDS-PAGE gel (A) and the Western blot membrane (B). Corresponds to Figure 3.

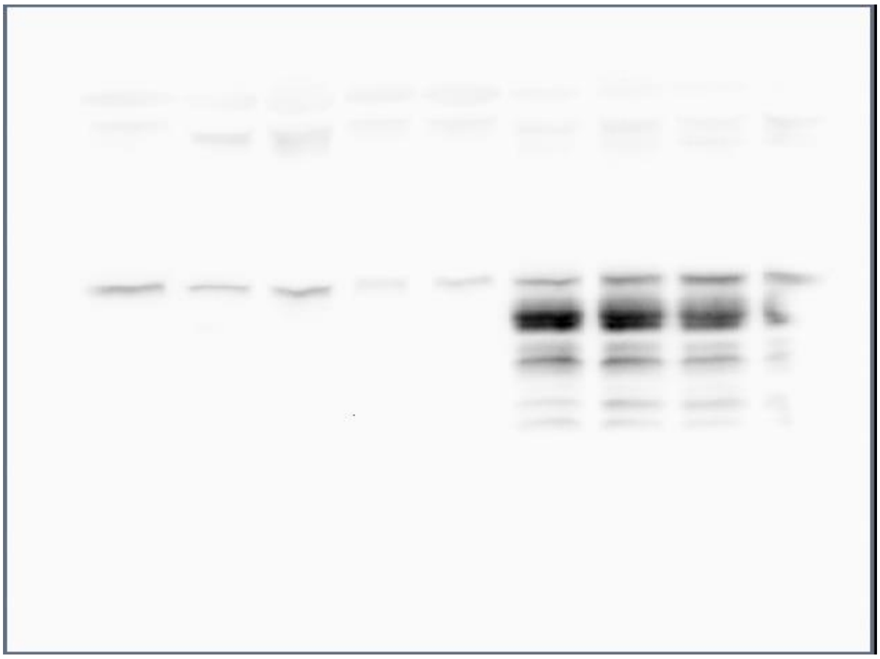
86s

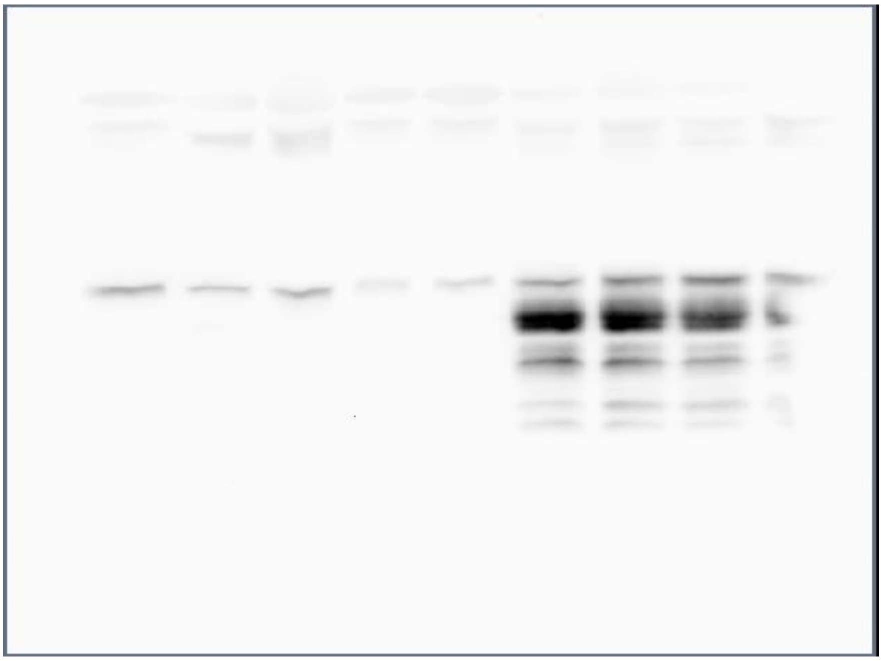
116s

Supplementary Figure 9. Series of images for hGMCSF Western blotting. Corresponds to Figure 3.

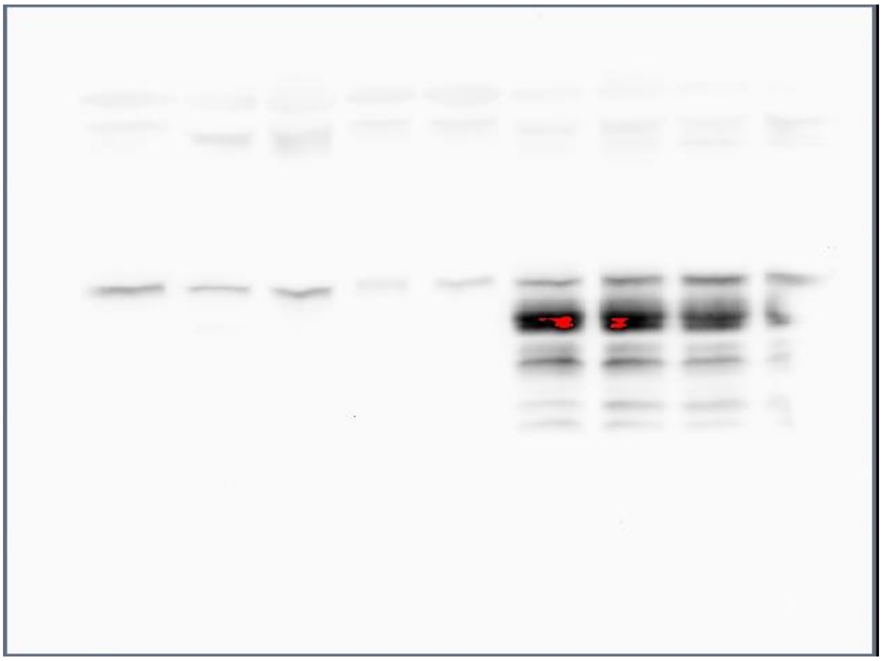

136s

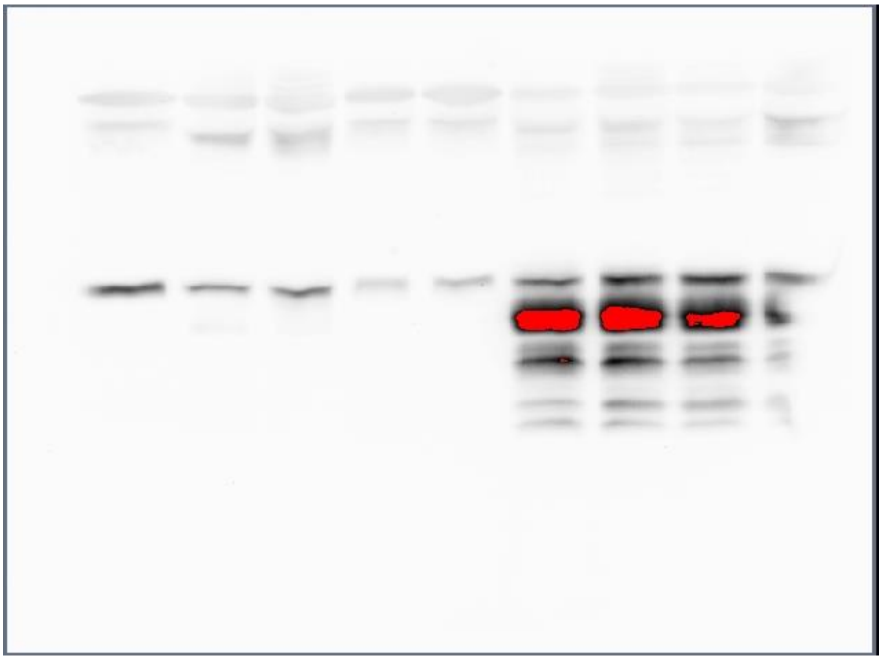

300s

Supplementary Figure 9 (continuation). Series of images for hGMCSF Western blotting. Corresponds to Figure 3.

SUPPLEMENTARY TABLES

Transgenic mouse line	Transferred blastocysts (% of injected)	Strain of host blastocysts	Recipient females	Pups born (%)	Chimeras born (%)	Chimerism % (No. of animals)	Male chimeras (%)	Fertile male chimeras (%)	Transgenic pups (of all agouti)
Insertion ES INS-GM	30/35 (85,7%)	CD-1	2	13 (43,3%)	9/13 (69,2%)	>80% (0) 30-80% (1) <30% (8)	7 (77,8%)	6 (85,7%)	20/41 (49%)
Replacement ES REP-GM	18/19 (94,7%)	B6D2F1	1	9 (50%)	9/9 (100%)	>80% (7) 30-80% (2) <30% (0)	7 (77,8%)	6 (85,7%)	26/57 (46%)

Supplementary Table 1. Germline transmission analysis for fertile male chimeras.

Supplementary Table 2. Germline transmission analysis for individual fertile male chimeras.

Transgenic mouse line	Fertile male chimera (individual number)	Chimerism %	Total pups born	Agouti pups (%)
Insertion ES INS-GM	1	<30%	61	0
	2	<30%	36	0
	3	40%	41	41 (100%)
	4	<30%	57	0
	5	<30%	40	0
	6	<30%	39	0
Replacement ES REP-GM	1	90%	14	14 (100%)
	2	90%	52	0
	3	90%	21	0
	4	90%	25	25 (100%)
	5	90%	36	0
	6	90%	18	18 (100%)

Supplementary Table 3. ddPCR analysis of the hGMCSF/Csn1s1 chimeric transcripts.

Sample (mammary gland cDNA)	Concentration (GMxCsn1s1 vs Csn2)	Standard error
WT 1	0	-
WT 2	0	-
INS-GM hemi 1	0,000931	0,00066-0,00122
INS-GM hemi 2	0,000793	0,00055-0,00093
INS-GM homo 1	0,00396	0,00348-0,00423
INS-GM homo 2	0,00588	0,00447-0,00657

Supplementary Table 4. Primers used in the project.

Primer name	Sequence 5'-3'	PCR experiment / product length (bp)
Csn1s1 forward primer	TGTAGTGGATCAGGCACTGG	Expression ddPCR Csn1s1 156
Csn1s1 reverse primer	TCCTTGGAGACAATGGGCTT	
Csn1s1 probe	HEX-CCAGTTCTCTGTTCAGCCCTTCCCACA-BHQ2	
Csn2 forward primer	AGGACTTGACAGCCATGAAGG	Expression ddPCR Csn2 125
Csn2 reverse primer	ATGTTCAACAGATTCCTCACTGGA	
Csn2 probe	FAM-ATCCTCGCCTGCCTTGTGGCCCTTGC-BHQ1	
GMxCsn1s1 forward primer	GACTTTCTGCTTGTCATCCCC	Expression ddPCR hGMCSF/Csn1s1 196
GMxCsn1s1 reverse primer	GTTGTTGACGAATTCCTGATTTAGG	
GMxCsn1s1 probe	HEX-CAGTCAAACTCAGCAACAGCATAGCAGCAG-BHQ2	
hGMCSF forward primer	AATGTTTGACCTCCAGGAGCC	Expression ddPCR hGMCSF 164
hGMCSF reverse primer	TCTGGGTTGCACAGGAAGTT	
hGMCSF probe	HEX-TGTACAAGCAGGGCCTGCGGGGCAG-BHQ2	
Rpl4 forward primer	GGCTGCTTCCCTCAAGAGTA	Expression ddPCR Rpl4 121
Rpl4 reverse primer	AATCTTCTTGCGTGGTGCTC	
Rpl4 probe	FAM-AGCCCAGAAATCCAAAGAGCCCTCC-BHQ1	
hGMCSF copy forward primer	TCTGGCAGGACTTTCCTCTG	CNV ddPCR hGMCSF 146
hGMCSF copy reverse primer	AGAAGGAGTGGAGTCAAGGC	
hGMCSF copy probe	HEX-CCCTTCCCGCAGGAAGGAGTGCC-BHQ2	
Emid1 copy forward primer	GCCAGGACTGGGTAGCAC	CNV ddPCR Emid1 79
Emid1 copy reverse primer	AGGAGGCTCCTGAATTTGTGACAAG	
Emid1 copy probe	FAM-CCTGGGTCATCTGAGCTGAGTCC-BHQ1	
ES-5H-F	TTGGCTGAGGTTAATTAGCTGAG	Genotyping both GM 5'-border 781
ES-5H-R	GTGCAGAGATGCTGCAGGC	
Seq_GMCSF_end_2_F	GGAAACTTCCTGTGCAACCCAG	Genotyping INS-GM 3'-border 1199
ES-3H-R	AGTTCTTGAAGGGCAGAGTATGG	
Seq_GMCSF_end_2_F	GGAAACTTCCTGTGCAACCCAG	Genotyping REP-GM 3'-border 1206
ES-3H-KOIN-R	CTGATATACACACATCATTCCTGGT	
Seq_5H_end_F	GCGCATAACTAAGCATCTTATGCT	Genotyping Csn1s1 283
Csns1-ATG-seq-R	GGTAGGAGTCTCGGGTGTAA	
Amp-check-F	CCCGGCGTCAATACGGGATAATAC	Genotyping backbone 5'-border 650
Seq_5H_beg_R	AAGATGGGGAAGAGAATCAGTC	
Clon-GFP-F	GAAAGACCCCACCTGTAGGT	Genotyping backbone 5'-border alt 704
ES-5H-R	GTGCAGAGATGCTGCAGGC	
Seq_GMCSF_end_2_F	GGAAACTTCCTGTGCAACCCAG	Genotyping backbone

BackboneFV-3H-R	GGAAAGCTTGCATGCAGGCCTC	3'-border 1147
ATG1 OffT1 F	TGCAGGAGAACCAGACAAGC	Off-target 1 Sanger 641
ATG1 OffT1 R	GGATCAGGATGAGCTGTGGC	
ATG1 OffT2 F	TCCATCAGGGTCAAAACCGAG	Off-target 2 Sanger 701
ATG1 OffT2 R	CAGAGCTGTGAGGACCGTTG	
KO1 OffT1 F	GGAGGTCACAGCACCAGTAC	Off-target 3 Sanger 465
KO1 OffT1 R	CCTGCCTCGAGACGAGATCA	
KO1 OffT2 F	GTCCGTATCACAGGCAGACG	Off-target 4 Sanger 765
KO1 OffT2 R	CTGCTCGAAGTCAGTGCTGG	

References

Hsu PD, Scott DA, Weinstein JA, Ran FA, Konermann S, Agarwala V, Li Y, Fine EJ, Wu X, Shalem O, Cradick TJ, Marraffini LA, Bao G, Zhang F. DNA targeting specificity of RNA-guided Cas9 nucleases. Nat Biotechnol. 2013 Sep;31(9):827-32. doi: 10.1038/nbt.2647.
